# Aminopeptidase secreted by *Chromobacterium sp*. Panama inhibits dengue virus infection by degrading the E protein

**DOI:** 10.1371/journal.pntd.0006443

**Published:** 2018-04-25

**Authors:** Raúl G. Saraiva, Jingru Fang, Seokyoung Kang, Yesseinia I. Angleró-Rodríguez, Yuemei Dong, George Dimopoulos

**Affiliations:** W. Harry Feinstone Department of Molecular Microbiology and Immunology, Bloomberg School of Public Health, Johns Hopkins University, Baltimore, MD, United States of America; The Pennsylvania State University, UNITED STATES

## Abstract

Dengue virus (DENV) is the most prevalent and burdensome arbovirus transmitted by *Aedes* mosquitoes, against which there is only a limited licensed vaccine and no approved drug treatment. A *Chromobacterium* species, *C*. *sp*. Panama, isolated from the midgut of *A*. *aegypti* is able to inhibit DENV replication within the mosquito and *in vitro*. Here we show that *C*. *sp*. Panama mediates its anti-DENV activity through secreted factors that are proteinous in nature. The inhibitory effect occurs prior to virus attachment to cells, and is attributed to a factor that destabilizes the virion by promoting the degradation of the viral envelope protein. Bioassay-guided fractionation, coupled with mass spectrometry, allowed for the identification of a *C*. *sp*. Panama-secreted neutral protease and an aminopeptidase that are co-expressed and appear to act synergistically to degrade the viral envelope (E) protein and thus prevent viral attachment and subsequent infection of cells. This is the first study characterizing the anti-DENV activity of a common soil and mosquito-associated bacterium, thereby contributing towards understanding how such bacteria may limit disease transmission, and providing new tools for dengue prevention and therapeutics.

## Author summary

The global burden of dengue cannot be overstated, and lack of effective control measures, besides avoiding contact with the mosquito vector, creates an urgent need to develop new strategies to control the disease. Here we elucidate the mechanism by which a natural soil bacterium inhibits DENV infection. *Chromobacterium sp*. Panama secretes a protease that is able to undermine the integrity of the viral particles, preventing their attachment and thus infection of host cells. This study contributes towards understanding how natural bacteria of the vector gut may limit disease transmission, and provides new candidate tools for the development of dengue transmission-blocking and therapeutic agents.

## Introduction

Dengue virus (DENV) is arguably the most prominent arboviral threat to humans in tropical areas, with 3.6 billion people at risk of infection and 100 million people symptomatically infected annually [1]. All four (1–4) DENV serotypes are able to cause significant disease, ranging from dengue fever to dengue hemorrhagic fever and dengue shock syndrome, these latter most often upon secondary infection [2]. The lack of approved drug regimens directly against the virus and the limited license of the only approved vaccine make preventing bites from the *Aedes* mosquito vector the most effective way of blocking disease transmission thus far.

DENV is a single-stranded, positive-sensed RNA virus of the *Flaviviridae* family. The viral genome is encapsidated by the capsid (C) proteins, and enveloped by a lipid bilayer within which the envelope (E) proteins and the mature membrane (M) proteins are embedded [3]. Among these three viral structural proteins, the E protein is responsible for binding to cell surface receptors, therefore triggering receptor-mediated endocytosis and subsequent internalization of the virion [4].

Our previous work has established that *Chromobacterium sp*. Panama can inhibit DENV infection in *Aedes aegypti* when it colonizes the mosquito midgut, as well as *in vitro* infection of cells when the virus is previously incubated with this bacterium [5]. This *Chromobacterium* species was isolated from *A*. *aegypti* in Panama and has also been shown to limit the life-span of laboratory-reared *A*. *aegypti* and *A*. *gambiae*, as well as directly interfere with *P*. *falciparum* development and infection. While the anti-DENV mechanism(s) were unknown, the *in vitro* inhibition suggested it was mosquito-independent [5], and indicated possible production of *C*. *sp*. Panama-derived antiviral factor(s) with transmission-blocking and therapeutic potential.

In this study, we aimed to identify the anti-DENV factors produced by *C*. *sp*. Panama and to describe their mechanism of action. We employed a bioassay-guided fractionation approach to discover a secreted aminopeptidase as mediating *C*. *sp*. Panama’s anti-DENV activity through the degradation of the viral E protein, and thus preventing virus attachment to the host cell and subsequent infection.

## Results

### *C*. *sp*. Panama culture supernatant shows potent anti-DENV activity

**Fig 1:**
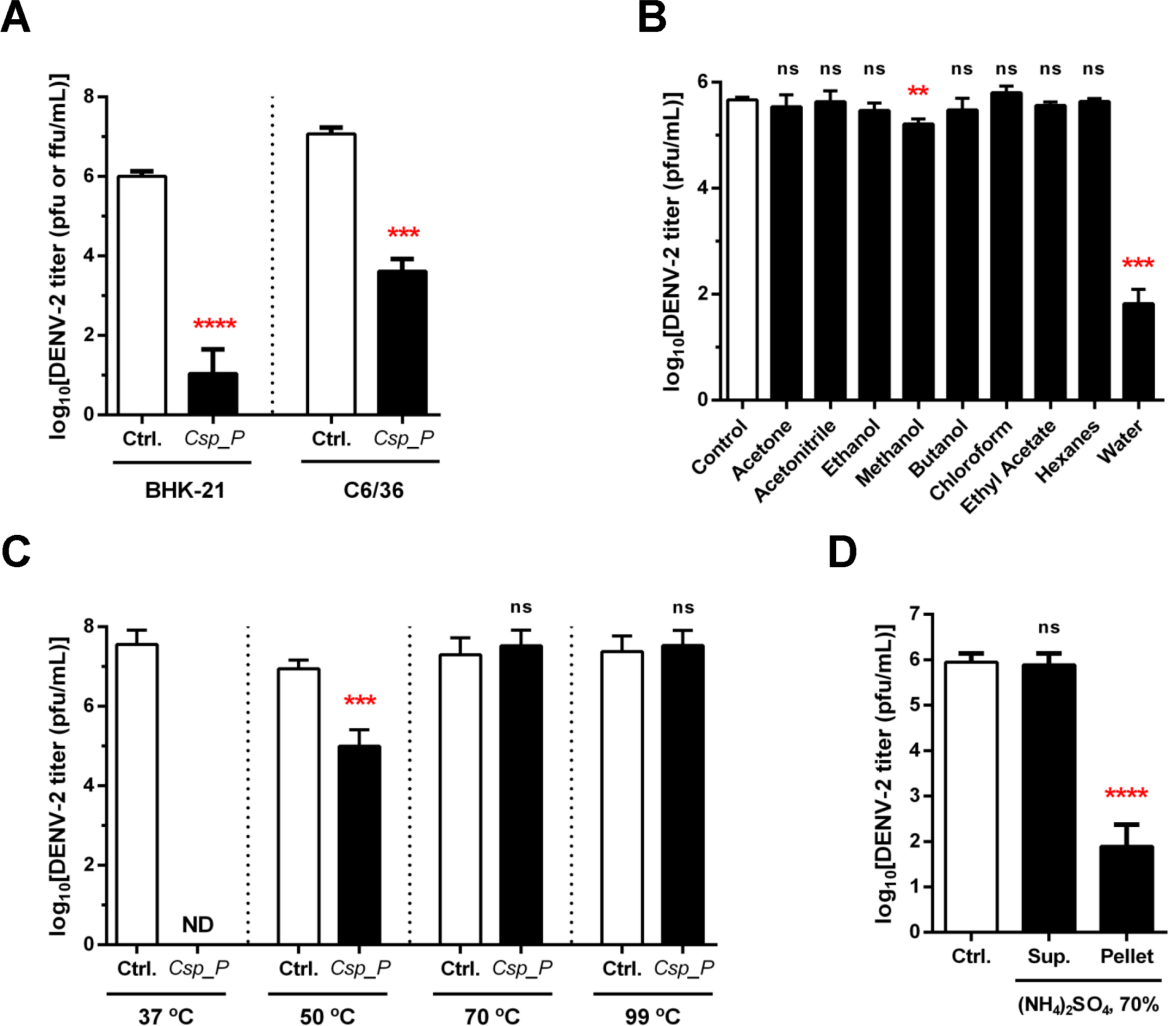
Anti-DENV activity of *Chromobacterium sp*. Panama is mediated by secreted proteinous factors. DENV virus titers: (**A**) in the presence or absence of culture supernatant of *C*. *cp*. Panama following infection in BHK-21 and C6/36 cells, as determined by plaque or focus forming assays, respectively; (**B**) in BHK-21 cells upon exposure to different solvent extracts of culture supernatants of *C*. *sp*. Panama; (**C**) in BHK-21 cells in the presence or absence of culture supernatant of *C*. *cp*. Panama incubated for 1 hour at the indicated temperatures; and (**D**) upon exposure to protein extract of the culture supernatant of *C*. *sp*. Panama prepared by 70% ammonium sulfate precipitation (Pellet), the desalted supernatant of that extraction (Sup.) or control buffer (0.1M Tris-HCl). Significance determined using unpaired t-tests; (ns, not significant; *p < 0.05; **p < 0.01; ***p < 0.001). Raw underlying data available in S2 Table.

In our previous work we explored the *in vitro* anti-DENV activity of *C*. *sp*. Panama by incubating unfiltered bacterial cultures with DENV particles, removing the bacterial cells only prior to assessing infection in mammalian cells or mosquito cells [5]. To understand if live bacteria are required to impair DENV infection, *C*. *sp*. Panama cultures were first filtered through a 0.22 μm membrane and only then mixed with the DENV viral stock. DENV replication in both BHK-21 cells (mammalian) and C6/36 cells (mosquito) was reduced by 3–5 logs (BHK-21: *p* < 0.0001, C6/36: *p* = 0.0009) upon treatment with this sterile supernatant when compared to the control (Fig 1A). This indicates that the *in vitro* anti-DENV activity displayed by *C*. *sp*. Panama could be attributed to secreted or otherwise released mediators and that live bacteria are not required to impair DENV infection. Similar level of *C*. *sp*. Panama-mediated anti-DENV activity in both cell lines suggests that the target of such bacteria-derived factors are likely to be viral proteins or RNA, or host factors that are conserved across species [6].

### Anti-DENV activity is associated with *C*. *sp*. Panama-produced proteinous factor(s)

To understand the nature of the *C*. *sp*. Panama-produced factors that influence DENV infection, we first partitioned the *C*. *sp*. Panama supernatant with commonly used organic solvents, both miscible and immiscible in water. *C*. *sp*. Panama molecules soluble in acetone, acetonitrile, butanol, chloroform, ethanol, ethyl acetate and hexanes showed no anti-DENV activity (Fig 1B). Even though methanol partially extracted molecules with anti-DENV properties (< 1-log reduction in DENV2 titer compared to control, *p* = 0.002), water was the only solvent that was able to retain a significant majority of the activity observed in the initial extract (4-log reduction, *p* = 0.0002), providing early evidence that the relevant *C*. *sp*. Panama-produced factors are likely charged or otherwise hydrophilic biomolecules, such as most proteins or sugars, and not lipidic in nature.

Next, we assessed the thermostability of such factors, concluding that 1 hour incubation at 50°C partially inactivated (2-log reduction in DENV2 titer compared to control, *p* = 0.0005) the level of anti-DENV activity of samples handled at room temperature (as before) or 37°C, for which no viral replication was detected (Fig 1C). Incubation at 70 or 99°C for 1 hour resulted in total loss of activity (Fig 1C). Taken together, these data strongly suggest that the *C*. *sp*. Panama-produced anti-DENV factors represent hydrophilic proteins, given the very strong preference for the aqueous solvent–the only that would ensure proper protein folding [7,8]. The dramatic loss of activity at 50°C and above would not be as readily expected from sugars or other small metabolites.

To further test our hypothesis, we used ammonium sulfate precipitation [9] to create protein extracts of the *C*. *sp*. Panama supernatant. The resulting protein suspension carried all the anti-DENV activity (4-log reduction in DENV titer compared to control, *p* < 0.0001, Fig 1D), whereas the supernatant, after appropriate desalting, was ineffective in reducing DENV infectivity *in vitro*. This result clearly indicates that proteinous factors, such as peptides, proteins or protein complexes, are the likely mediators of *C*. *sp*. Panama’s anti-DENV activity.

### *C*. *sp*. Panama-produced proteins inhibit DENV attachment to host cells

**Fig 2:**
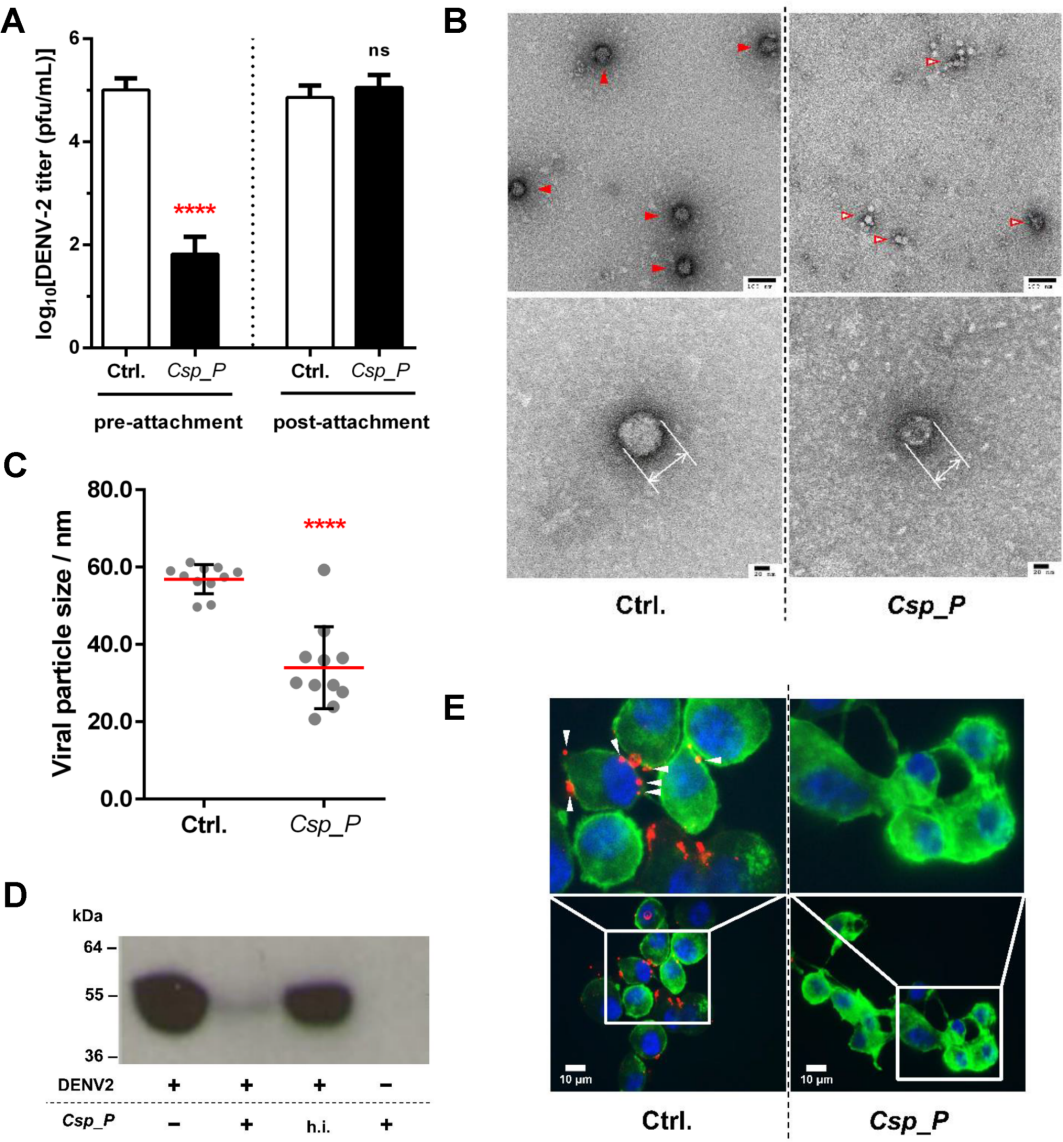
Protein extracts of culture supernatants of *C*. *sp*. Panama inhibit DENV entry by mediating degradation of the E protein. (**A**) DENV titers following exposure to a protein extract of the *C*. *sp*. Panama culture supernatant before or after viral attachment to BHK-21 cells; significance determined using unpaired t-tests. Raw underlying data available in S2 Table. (**B**) Representative negative stained transmission EM images of control DENV particles (arrow head) and those treated with *C*. *sp*. Panama culture supernatant protein extract (empty arrow head); low magnification: 93,000X, high magnification: 245,000X. (**C**) Size distribution of the viral particles as measured from the transmission EM images collected in B; mean and standard deviation indicated; significance determined using unpaired t-test. (**D**) Western blot (anti-E) of DENV proteins when exposed to the *C*. *sp*. Panama culture supernatant protein extract. (**E**) Fluorescence microscopy showing DENV attachment to BHK-21 cells in the presence of the protein extract of the *C*. *sp*. Panama. Cells were fixed with 4% PFA, stained for DNA (DAPI-blue), and F-actin (Phalloidin-green). Membrane-bound DENV (arrow head) were immunostained with 4G2 anti-E antibody (Red). ns, not significant; *p < 0.05; **p < 0.01; ***p < 0.001.

Attachment of viral particles to host cells is the first step that initiates virus entry, and thus precedes all the following steps necessary for viral replication. To understand if our *C*. *sp*. Panama proteinous extract exerts its anti-DENV effect by interfering with viral attachment, we performed neutralization assays pre- and post-attachment using low temperature to arrest receptor-mediated endocytosis as before [10]. In the pre-attachment assay, we incubated DENV virions with *C*. *sp*. Panama proteins at room temperature and then exposed this mixture to pre-chilled cells at 4°C to allow for viral attachment without endocytosis. Unbound virions were washed off, and attached virions were allowed to complete entry and infection in host cells at 37°C. In the post-attachment assay, DENV virions were first adsorbed to pre-chilled cells at 4°C and, only after extensive washing, *C*. *sp*. Panama proteins were added to treat attached virions. Our results showed a significant 3-log decrease in DENV titers compared to the control (*p* < 0.0001) when DENV was exposed to the *C*. *sp*. Panama proteins before attachment (Fig 2A). In contrast, *C*. *sp*. Panama proteins failed to inhibit DENV replication when attachment had already occurred (Fig 2A), indicating that the *C*. *sp*. Panama-produced anti-DENV factor(s) is likely to either interfere with the viral particle itself and/or prevent its attachment to host cells.

### *C*. *sp*. Panama interferes with DENV attachment by mediating degradation of the E protein

To investigate whether *C*. *sp*. Panama proteins affect viral particles, we incubated the protein extract with purified DENV2 virions and processed the samples for transmission electron microscopy (Fig 2B). The DENV mature particle has a diameter of 50–60 nm [11,12] and, fully consistent with that, we obtained a measurement of 56.7±1.1 nm for the diameter of the viral particles in our control sample in Tris-HCl, pH 7.2 (Fig 2C). Upon treatment with *C*. *sp*. Panama proteins in the same buffer, however, the viral particles exhibited a significantly different (*p* < 0.0001) diameter of 34.0±3.2 nm (Fig 2C). The DENV E protein occupies a 180–280 Å (*i*.*e*. 18–28 nm) radius of the particle, as shown by analysis of a class of virus with loose or absent E protein shell with diameter of ~36 nm [12]. As such, we hypothesized that treatment with our bacterial protein extract compromised the integrity of the DENV particle E protein coating layer. This is in agreement with our previous finding that viral attachment is compromised, as the E protein is a crucial mediator of viral attachment to, and fusion with, host cells [13,14].

To test this hypothesis, we exposed DENV to the *C*. *sp*. Panama protein extract as before and analyzed the presence of DENV E protein by Western blotting. As shown in Fig 2D, a significantly diminished signal for the E protein at the expected size of 53 kDa [14] is detected in the sample treated with *C*. *sp*. Panama protein extract, the same not being true for when DENV is treated with a heat-inactivated *C*. *sp*. Panama protein extract. Our immunofluorescence microscopy results were consistent with this finding by failing to detect attached virions to BHK-21 cells with another anti-E protein antibody (4G2, ATCC; Fig 2E). However, as the cells were washed prior to staining, it is possible to interpret that any remaining bound virus (as hinted in Fig 2A) was simply below the detection limit for this technique. Nonetheless, the Western blotting data show E protein degradation upon treatment with *C*. *sp*. Panama proteins, pointing to a potential *C*. *sp*. Panama-secreted protease as the most likely factor responsible for this effect.

### Secreted proteases are present in active anti-DENV protein fraction

**Fig 3:**
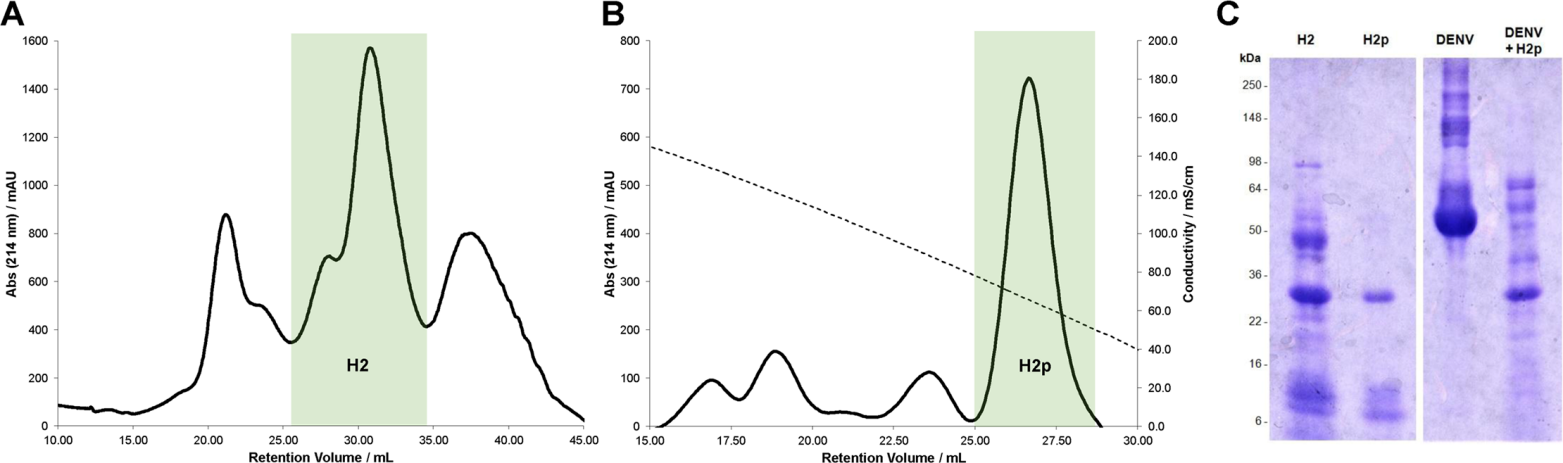
Purification of the anti-DENV proteins of *C*. *sp*. Panama. (**A**) Hydrophobic interaction FPLC chromatogram (absorbance at 214 nm) of the protein extract of *C*. *sp*. Panama. Fraction H2 highlighted. Column: HiTrap FF 1 mL. Flow rate: 1 mL/min. Gradient elution from 1.5 to 0 M ammonium sulfate in 50 mM sodium phosphate (pH 7.0). (**B**) Hydrophobic interaction FPLC chromatogram (absorbance at 214 nm) of H2 from A. Fraction H2p highlighted. Column: HiTrap HP 1 mL. Flow rate: 1 mL/min. Gradient elution from 1.5 to 0 M ammonium sulfate in 50 mM sodium phosphate (pH 7.0); conductivity (mS/cm) showed by dashed line. (**C**) SDS-PAGE protein gel of H2, H2p, DENV2 protein extract and co-incubation of this extract with H2p.

In a parallel approach, we also sought to further partition the *C*. *sp*. Panama culture supernatant protein extract in search of the causative agent for the anti-DENV activity. For this purpose, we employed hydrophobic interaction chromatography (HIC) to separate proteins based on their content in hydrophobic amino acids. Following an initial fractionation in butyl Sepharose under a salt gradient, we were able to obtain a discrete fraction (H2, Fig 3A) that preserved the anti-DENV properties. This fraction was further purified by a second round of fractionation where conductivity along the gradient was monitored to match ~60 mS/cm, resulting in the collection of fraction H2p (Fig 3B). SDS-PAGE gels of these fractions revealed that fraction H2p is considerably less diverse than H2, with the exception of a band at approximately 30 kDa and other small proteins or peptides below 16 kDa (Fig 3C). Most importantly, this fraction retained proteolytic activity, as shown in Fig 3C, by its ability to degrade DENV proteins, including the E protein whose signal can be seen at ~53 kDa band.

Given the low complexity of the H2p fraction, we next chose to perform a proteomic analysis by mass spectrometry to understand which *C*. *sp*. Panama proteins were contained within this sample and the results are summarized on Table 1 (list of protein hits presented in S1 Table). Of the eight detected proteins, two (CSPP0261 and CSPP0262) were of particular relevance given their assignment as proteolytic. The neutral protease (CSPP0261) and the aminopeptidase (CSPP0261) are encoded in tandem in the *C*. *sp*. Panama genome, and both are assigned to be secreted according to the most closely related UniProt reviewed entries: P14756 from *Pseudomonas aeruginosa* for CSPP0261 and Q01693 from *Vibrio proteolyticus* for CSPP0262, making them ideal candidates for subsequent studies.

**Table 1:** Proteins recovered from active anti-DENV fraction of *C*. *sp*. Panama.

ID	Name	EC number	GO—biological process	MW / kDa
0261	Neutral protease	3.4.24.25	proteolysis	53.4
0262	Aminopeptidase	3.4.11.10	proteolysis	44.7
1115	Ferredoxin—NADP(+) reductase	1.18.1.2	oxidation-reduction process	29.5
1272	Arginine deiminase	3.5.3.6	protein citrullination	46.1
1432	Beta-N-acetylhexosaminidase	3.2.1.52	carbohydrate metabolic process	73.1
3050	Phosphoserine transaminase	2.6.1.52	L-serine & pyridoxine biosynthetic process	40.3
4002	Bacterioferritin	1.16.3.1	cellular iron ion homeostasis	18.5
4290	Dihydrolipoyl dehydrogenase	1.8.1.4	oxidation-reduction process	50.0

### Aminopeptidase secreted by *C*. *sp*. Panama mediates its anti-DENV activity

**Fig 4:**
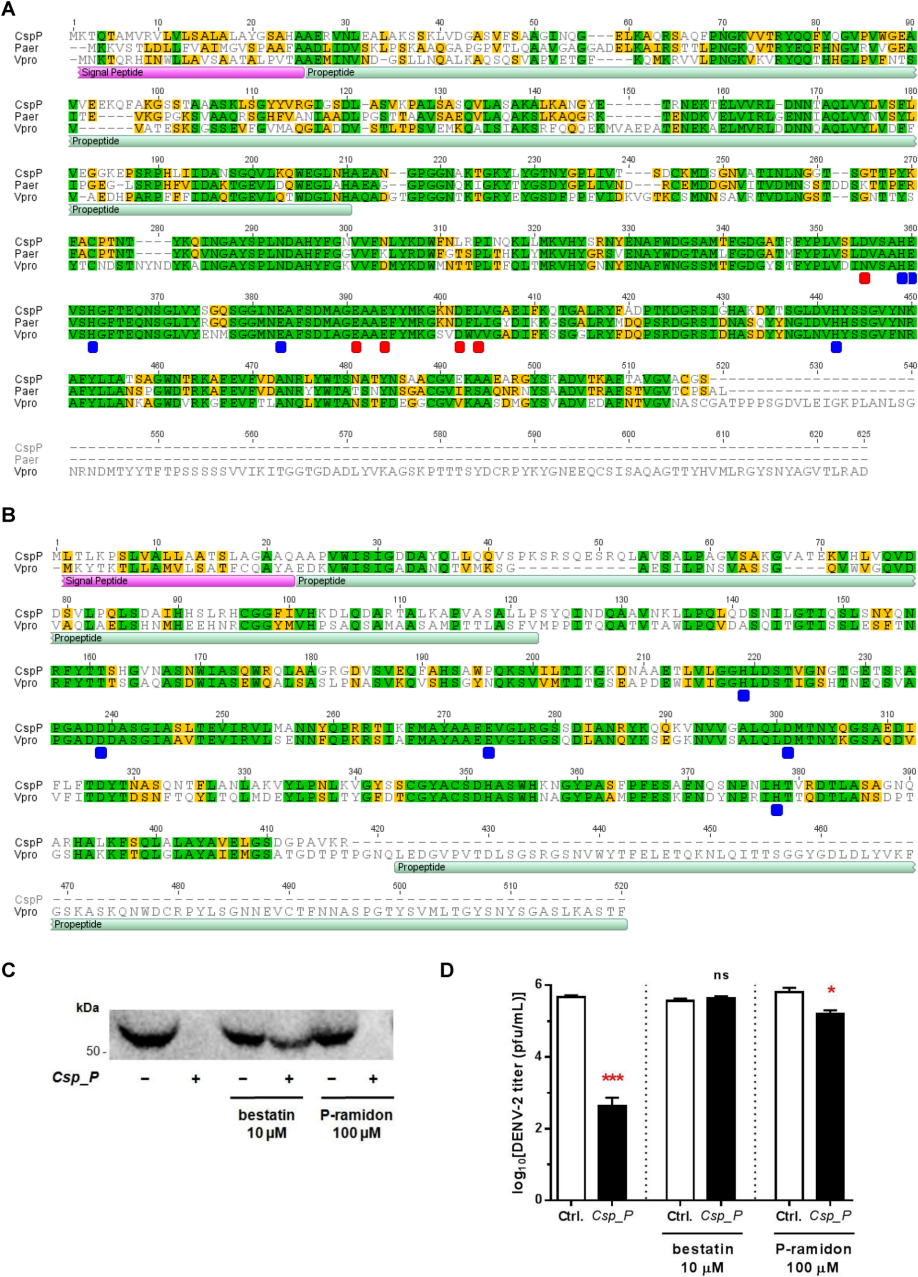
Aminopeptidase secreted by *C*. *sp*. Panama induces DENV E protein degradation, thereby inhibiting viral entry. (**A**) Protein sequence alignment of CSPP0261 and orthologs in *Pseudomonas aeruginosa* (Paer, UniProt: P14756) and *Vibrio proteolyticus* (Vpro, UniProt: Q00971). Conserved active and metal binding sites highlighted in blue (in both orthologs) and red (in *P*. *aeruginosa* ortholog). (**B**) Protein sequence alignment of CSPP0262 and ortholog in *Vibrio proteolyticus*. Conserved active and metal binding sites highlighted in blue (Vpro, UniProt: Q01693). (**C**) Western blot (anti-E-DENV) of DENV2 proteins when exposed to the culture supernatant of *C*. *sp*. Panama with or without supplementation with 10 μM bestatin or 100 μM phosphoramidon (P-ramidon). (**D**) DENV titers in BHK-21 cells following incubation of the virus with the *C*. *sp*. Panama culture supernatant with or without supplementation with 10 μM bestatin or 100 μM phosphoramidon (P-ramidon); significance determined using unpaired t-tests (ns, not significant; *p < 0.05; **p < 0.01; ***p < 0.001). Raw underlying data available in S2 Table.

Having putatively narrowed down the anti-DENV activity of the *C*. *sp*. Panama culture supernatant to two proteases, we aimed at a better understanding of these two enzymes by comparing their sequence to similar proteins already discussed in the literature. For the neutral protease CSPP0261, BLAST analysis returned significant similarity to the *P*. *aeruginosa* pseudolysin (59.3% pairwise identity) and the *V*. *proteolyticus* vibriolysin (49.7% pairwise identity), two secreted zinc metallopeptidases of the M4 family that form a divergent branch within the clan [15]. They have been described as having a nutritional role by scavenging amino acids for the bacteria, as well as a virulence role in pathogenic species, either by directly mediating the destruction of tissue or indirectly by interfering with host defense mechanisms or the normal function of host proteases [16]. Sequence alignment showed that the neutral protease CSPP0261 conserves the active and metal binding sites of these enzymes (Fig 4A), and that it lacks the secondary C-terminal catalytic domain of vibriolysin [17].

For the aminopeptidase CSPP0262, the only matching fully reviewed entry in UniProt corresponded to the *Vibrio* aminopeptidase (39.6% pairwise identity) from *V*. *proteolyticus*, also a secreted zinc metallopeptidase but belonging to the M28 family as it binds two zinc ions that co-catalyze the proteolytic reaction [18]. This enzyme removes free N-terminal amino acids of peptides or proteins that are hydrophobic and large (preferably), basic or proline, but never aspartyl, glutamyl or cysteic acid residues; this capability has been harvested in biotechnological settings namely in the production of the antiviral and anticancer agent interferon alfa-2b [18,19]. Sequence alignment to the aminopeptidase CSPP0262 showed perfect agreement in all the annotated active metal binding sites for the *V*. *proteolyticus* ortholog (Fig 4B); CSPP0262 lacks the C-terminal propeptide present in the *Vibrio* aminopeptidase for which, however, no function has been assigned [20,21].

Of note, both proteases contain a conserved signal peptide sequence potentially also governing their secretion by the *Chromobacterium* species. Also conserved is an N-terminal propeptide that has been shown for both ortholog proteins to be crucial for correct folding of the mature protein, having the ability to inhibit its activity until secretion to prevent intracellular damage [16,20,21]. After processing, both *C*. *sp*. Panama enzymes would assume an active form of ~32 kDa, consistent with our observation that our proteolytically active H2p fraction is enriched with a protein of this size (Fig 3C). A difference between the two peptidase families, however, is their susceptibility to inhibitors: phosphoramidon is known to inhibit the activity of the M4 proteases [22] whereas bestatin has been described as an inhibitor of the M28 peptidase [18]. In an attempt to determine which of these enzymes is responsible for the anti-DENV activity of *C*. *sp*. Panama, we grew the bacteria in media supplemented with each of these inhibitors and evaluated the culture supernatant for its ability to degrade the DENV2 E protein.

As shown in Fig 4C, treatment with 10 μM bestatin, and not 100 μM phosphoramidon, was able to revert E protein degradation by the *C*. *sp*. Panama culture supernatant. This indicates that the activity of the CSPP0262 aminopeptidase is crucial for digestion of the E protein, with minimal contribution of CSPP0261. These results are replicated when assaying the effect of these inhibitors on DENV titers by plaque assay with a total ablation of anti-DENV activity in the presence of bestatin (Fig 4D), but a more prominent role can now be postulated for CSPP0261. Even though supplementation of bacterial growth media with phosphoramidon is not able to fully counteract the inhibitory activity of the *C*. *sp*. Panama culture supernatant, it is apparent that this inhibitor aids in rescuing the phenotype. Taken together, the results of our analyses point to the CSPP0262 aminopeptidase as the causative agent of the anti-DENV activity by *C*. *sp*. Panama, and support a facultative role of the CSPP0261 neutral protease in the process.

## Discussion

The anti-DENV activity of *C*. *sp*. Panama was further investigated in the present work, building on the initial observation that this bacterium can inhibit viral replication *in vivo* and *in vitro* [5]. First, we validated that the activity is mediated by a secreted factor and does not require live bacteria; retention of activity by protein extracts of the culture supernatant further indicated that a protein was responsible for the effect. Then, we demonstrate that the *C*. *sp*. Panama protein extract interfered with viral attachment by promoting the degradation of its E protein. Successive fractionation of this extract allowed for the isolation and identification of two tandemly expressed proteases, CSPP0261 and CSPP0262, that appear to function somewhat synergistically towards the anti-DENV activity of *C*. *sp*. Panama, as indicated by experiments conducted with specific inhibitors to each of the peptidases.

A mechanism for this synergy can be speculated based on studies conducted with the ortholog enzymes expressed and secreted by *V*. *proteolyticus*. As mentioned earlier, the CSPP0262 aminopeptidase contains an N-terminal propeptide that has been shown in the *Vibrio* ortholog to be inhibitory towards its proteolytic activity until it is removed. This cleavage can happen autocatalytically [23], but a role has been proposed for vibriolysin as the biologically relevant activating peptidase [24]. Vibriolysin and the *Vibrio* aminopeptidase are co-expressed [24], as also CSPP0261 and CSPP0262 appear to be, which further validates this observation. *P*. *aeruginosa* also secretes an aminopeptidase belonging to the M28 family that is processed to its mature form by pseudolysin [25], but BLAST analysis revealed weak identity between this aminopeptidase and CSPP0262 (31% identity; 24% query cover). Given this, our findings manipulating this system with the different protease-specific inhibitors suggest an effector role for the CSPP0262 aminopeptidase, with the CSPP0261 neutral protease perhaps acting to facultatively cleave and activate the aminopeptidase. This is compatible with the observation made in *V*. *proteolyticus* that, even though the aminopeptidase is able to be activated by autocatalysis, that route is less efficient when compared to the activation by the respective neutral protease [23].

Ours is the first study characterizing the mechanisms of the anti-DENV activity of a bacterium that was originally isolated from the *Aedes* mosquito midgut. It is improbable that *C*. *sp*. Panama has evolved to secrete the CSPP0262 aminopeptidase to benefit from its antiviral properties; a more likely scenario has this aminopeptidase as having a primordial role in digestion and nutrient scavenging similar to what has been put forward for the ortholog in *V*. *proteolyticus*. Interestingly, while the highly proteolytic environment of the blood-fed mosquito midgut [26] does not significantly impact DENV infection, the *C*. *sp*. Panama-produced protease does, thereby representing a potent anti-DENV effector molecule. This knowledge could be used towards developing transmission-blocking strategies, based on the presence of *C*. *sp*. Panama itself in the midgut, or through transgenic or paratransgenic expression of the anti-DENV protease in the midgut tissue [27,28]. While it is not unconceivable that DENV could acquire mutations in its E protein that render the activity of the aminopeptidase from *C*. *sp*. Panama ineffective, we can hypothesize that that would come at a high fitness cost for the virus given the high specialization of this envelope protein and its sequential role in both attachment and entry.

As such, a potent anti-DENV protease could also be developed into a therapeutic agent to treat viral infection in humans. Often overlooked as a class of therapeutics, proteases have been approved by the U.S. FDA to treat conditions such as hemophilia, acute myocardial infarction or muscle spasms since the 1980s [29,30]. While their antiviral potential has only been explored in topical applications against cold-causing rhinoviruses and, more recently, herpes simplex virus [31], the great amount of research on alternative-to-parenteral delivery methods for biologics brings promise on the ability to deliver therapeutic proteins through non-invasive routes such as orally or intranasally in the near future [32–34]. Other potential hindrances relate to their propensity for immunogenicity in the form of anti-drug antibodies and the lack of specificity of their proteolytic activity as measured by the number of putative substrates, but these obstacles have also been extensively addressed and strategies developed to overcome them [35–37]. For example, the botulinum toxins are also zinc metalloproteases initially characterized from bacteria: significant advances since their introduction have allowed for new-generation drugs with reduced immunogenicity and increased stability that can be absorbed transdermally [38,39]. The evaluation of the aminopeptidase from *C*. *sp*. Panama as an antiviral could benefit and be expedited from the progress made in this research arena. The potential versatile applications of *C*. *sp*. Panama aminopeptidase in dengue control warrants further basic and translational investigations.

## Methods

### Cell culture maintenance, virus propagation and bacterial growth

Cell lines were maintained and viral stocks prepared as previously described [40]. BHK-21 hamster kidney cells (ATCC CCL-10) were grown at 37°C, 5% CO_2_, in DMEM containing 10% fetal bovine serum (FBS), 1% penicillin- streptomycin and 5 μg/ml Plasmocin. C6/36 *Aedes albopictus* cells (ATCC CRL-1660) were grown at 32°C, 5% CO_2_, in MEM, with the same additives, except Plasmocin, plus 1% non-essential amino acids. C6/36 cells were used to propagate DENV2 New Guinea C strain; after 6 days post infection (dpi), cells were lysed by three successive freeze-thaw cycles and clarified by centrifugation at 1,500–2,000 *g* for 10 minutes, from which the soluble content was combined with cell supernatant as viral stock. This viral stock was aliquoted and stored at −80°C for later use. Unless otherwise indicated, *Chromobacterium sp*. Panama was grown in LB broth at 30°C for 72 hours at 250 rpm. Culture supernatants were obtained by filtering this preparation through a 0.22 μm syringe or disk filter.

### Dengue virus titration

Levels of DENV2 infection were assayed as before: by plaque assay in BHK-21 cells [40] or focus forming assay in C6/36 cells [41]. Both cell lines were seeded at ~8.4×10^5^ cells/well and then incubated at 37°C or 32°C overnight, respectively, before reaching 80–90% confluence. Cell monolayers were then incubated for 1 hour with untreated DENV2 viral stock or a 1:1 mixture of the stock with the different treatments after 1 hour of pre-incubation and covered with a 0.8% methylcellulose overlay DMEM medium with 2% FBS. After 5–6 dpi BHK-21 cell monolayers were fixed and stained with 1% crystal violet in methanol/acetone (1:1). For the focus forming assay, C6/36 cells were fixed with methanol/acetone (1:1) and blocked with 5% skim milk in PBS. The C6/36 monolayer was probed with mouse anti-flavivirus envelope protein antibody (4G2, ATCC; 1:1,000–1:2,000) followed by goat anti-mouse IgG-HRP (1:1,500), and developed with True Blue Peroxidase Substrate (KPL). In each case, the number of plaque or focus forming units (PFU, FFU) per ml was determined, and unpaired t-tests used to conclude on the significance of differences seen to the respective controls, correcting for multiple comparisons with the Welch’s method when appropriate.

### Solvent extractions of bacterial supernatants

For the water-immiscible solvents, *C*. *sp*. Panama supernatant was mixed with butanol, chloroform, ethyl acetate and hexanes, at 1:1 and the non-aqueous phase was recovered. For acetone, acetonitrile, ethanol, methanol and water, *C*. *sp*. Panama supernatants were desiccated in an Eppendorf Vacufuge and the resulting residue resuspended in the same volume of the different solvents for 4 hours under continuous shaking (1400 rpm). Particles that did not resuspend were then pelleted by 10 minutes centrifugation at 8,000 *g* in a table top centrifuge and the supernatants were recovered. All extracts were then desiccated in an Eppendorf Vacufuge and the resulting residue resuspended in the same volume of water for 4 hours under continuous shaking (1400 rpm). After being filtered through a 0.22 μm syringe filter, each extract was added 1:1 to the DENV2 stock and incubated at room temperature for 45 minutes, and infectivity was assayed by plaque assay in BHK-21 cells. Desiccated LB medium resuspended in water was used as negative control.

### Protein extract preparation

Ammonium sulfate, (NH_4_)_2_SO_4_, was slowly added to the *C*. *sp*. Panama supernatant until reaching 70% saturation. After rapid stirring at 4°C for 1 hour, proteins were pelleted down by centrifugation at 10,000 *g* for 20 minutes. The protein pellet was then gently resuspended in 0.1 M Tris-HCl pH 7.2 buffer and concentrated using an ultra-centrifugal filter unit (nominal molecular weight limit, 30 kDa; Amicon). Protein concentrations were determined by BCA assay (Pierce, Thermo Scientific). For comparison, the ammonium sulfate-rich supernatant was desalted and concentrated to the same extent.

### Pre- and post-virus attachment neutralization assays

The following protocols were adapted from the literature [10]. Pre-attachment: BHK-21 cell monolayers and reagents were cooled to 4°C for 1 hour. Virus and *C*. *sp*. Panama extract were incubated at 4°C for 1 hour, and then adsorbed to pre-chilled BHK-21 monolayers for another hour at 4°C. After adsorption, cell monolayers were washed 3 times with cold PBS, overlay medium was added and cells were incubated at 37°C for 5 days before fixing and staining for plaque detection.

Post-attachment: BHK-21 monolayers and reagents were pre-chilled to 4°C for 1 hour. Virus was added to cells and allowed to attach for 1 hour at 4°C. Unbound virus was washed off by washing twice with cold PBS. *C*. *sp*. Panama extract was then added to virus bound cells and incubated for another hour at 4°C. Following incubation, cell monolayers were washed once with cold PBS. Overlay medium was added and cells were incubated at 37°C for 5 days before fixing and staining for plaques.

### Transmission electron microscopy

For purification of viral particles, DENV2 stocks were pelleted in 8% w/v PEG 8000 at 14,000 *g* for 1 hour, further purified by 24% w/v sucrose cushion for 1 hour at 248,000 rpm (Beckman SW28 rotor) and separated in potassium tartrate-glycerol gradient 10–30% for 2 hours at 175,000 *g* (Beckman SW55Ti rotor), as described previously [42]. Pure DENV2 viral particles suspended in 1:1 v/v 0.1 M Tris-HCl (pH 7.2) or in the *C*. *sp*. Panama proteinous extract were incubated at room temperature for 1 hour and virus within the samples was sequentially inactivated in 4% paraformaldehyde in PBS for 30 minutes at 25°C followed by 30 minutes at 37°C. The samples in 0.08 M phosphate were then applied to a carbon grid, washed 3 times with 50 mM TBS and negatively stained with 1% w/v uranyl acetate 0.04% w/v trehalose. The grids were allowed to air-dry and images were acquired in a Hitachi 7600 TEM microscope at 80.0 kV.

### Protein gels and Western blotting

Viral stocks were incubated 1:1 v/v with the differentially treated *C*. *sp*. Panama culture supernatants, their protein extracts or control buffers for 1 hour at room temperature. For heat inactivation, the protein extract was incubated at 90°C for 1 hour prior to being added to the virus suspension. Samples were then incubated at 85°C for 2 minutes in SDS-containing loading buffer and ran on 4–20% tris-glycine gels under denaturing conditions. Protein bands were dry-transferred to a nitrocellulose membrane, followed by overnight blocking at 4°C in 5% skim milk 0.1% Tween 20 PBS. Membranes were probed with an anti-DENV2-E rabbit polyclonal (1:2,000) followed by anti-rabbit HRP-linked donkey (1:20,000, Amerhsam) antibodies for 1 hour each, with abundant wash with 0.1% Tween 20 PBS after each probing. Chemiluminescence signals were detected using Amersham ECL Prime Western Blotting detection reagents.

### Immunofluorescence microscopy

Immunostaining and fluorescence microscopy was conducted as described before [43], with adaptations. BHK-21 cells were seeded on a glass coverslip, grown at 37°C up to ~50% confluency, and incubated with DENV2 in the presence and absence of *C*. *sp*. Panama proteins for 1 hour at 4°C. Cells were washed with cold PBS and fixed with 4% paraformaldehyde-PBS for 15 minutes at room temperature. Fixed cells were blocked with 0.5% BSA in PBS for 1 hour at room temperature, washed with PBS and incubated with mouse 4G2 antibody (ATCC) for 2 hours at room temperature. Alexa Fluor 568 goat anti-mouse IgG (Invitrogen) was used as secondary antibody. Beta-actin was stained with Alexa Fluor 488 Phalloidin (Invitrogen) and nuclei were stained with DAPI. Samples were mounted with ProLong Gold antifade reagent and visualized on a Leica DM 2500 fluorescence microscope.

### Hydrophobic interaction chromatography

Fast protein liquid chromatography was performed using an ÄKTA Purifier system. Hydrophobic interaction FPLC was conducted on protein extracts of *C*. *sp*. Panama resuspended using HiTrap Butyl FF or Butyl HP columns (GE Healthcare Life Sciences) under gradient elution from 1.5 to 0 M ammonium sulfate in 50 mM sodium phosphate (pH 7.0) at constant 1 mL/min flow rate. Collected fractions were dialyzed using ultra-centrifugal filter units (nominal molecular weight limit, 30 kDa; Amicon) for solvent replacement as needed.

### Proteomics and protein sequence analysis

Fraction H2p in 0.1M Tris-HCl was submitted to the Johns Hopkins Medicine Mass Spectrometry Core for identification using iTRAQ as before [44]. Peptides obtained were searched against the theoretical proteomes of *Chromobacterium spp*. following trypsin digestion using Mascot (Matrix Science); Scaffold 4.8 (Proteome Softwares, Inc.) was used to validate protein identification at 99.0% protein probability threshold, 4 as the minimum number of peptides and 95% as the peptide probability threshold. Amino acid sequences of identified peptides and proteins were further searched against the *C*. *sp*. Panama theoretical proteome based on its genomic information (NCBI Identifier QARX00000000) for validation of the findings. Sequences of proteins of interest were subjected to basic local alignment tools (BLAST) against the UniProt database and further aligned to the closest related annotated entry using the Geneious v5.4 algorithm (Biomatters Limited).

### Protease inhibition

Bestatin and phosphoramidon (Sigma) were diluted in water and supplemented to LB at the indicated concentrations prior to inoculation of *C*. *sp*. Panama. Following 72 hours incubation at 30°C, bacterial culture supernatants were collected and either processed as before for Western blotting or evaluation of effect on DENV2 titer by plaque assay on BHK-21 cells.

## Supporting information

S1 Table Proteins recovered from active anti-DENV fraction of *C*. *sp*. Panama. Expanded version of Table 1 with sequence information.
(XLSX)

S2 Table Raw data for graphs in Figs 1, 2 and 4. (XLSX)
